# A novel thermotolerant l-rhamnose isomerase variant for biocatalytic conversion of d-allulose to d-allose

**DOI:** 10.1007/s00253-024-13074-w

**Published:** 2024-04-02

**Authors:** Sweety Sharma, Satya Narayan Patel, Sudhir P. Singh

**Affiliations:** 1 Center of Innovative and Applied Bioprocessing, Biotechnology Research and Innovation Council (Department of Biotechnology, Government of India), NABI Campus, SAS Nagar, Sector 81, Mohali, India 140306; 2 Indian Institute of Science Education and Research Mohali, SAS Nagar, Sector 81, Mohali, India 140306

**Keywords:** d-Allose, d-Allulose, l-Rhamnose isomerase, Metagenome

## Abstract

**Abstract:**

A novel l-rhamnose isomerase was identified and cloned from an extreme-temperature aquatic habitat metagenome. The deduced amino acid sequence homology suggested the possible source of this metagenomic sequence to be *Chloroflexus islandicus*. The gene expression was performed in a heterologous host, *Escherichia coli*, and the recombinant protein l-rhamnose isomerase (L-RI_M_) was extracted and purified. The catalytic function of L-RI_M_ was characterized for d-allulose to d-allose bioconversion. d-Allose is a sweet, rare sugar molecule with anti-tumour, anti-hypertensive, cryoprotective, and antioxidative properties. The characterization experiments showed L-RI_M_ to be a Co^++^- or Mn^++^-dependent metalloenzyme. L-RI_M_ was remarkably active (~ 80%) in a broad spectrum of pH (6.0 to 9.0) and temperature (70 to 80 °C) ranges. Optimal L-RI_M_ activity with d-allulose as the substrate occurred at pH 7.0 and 75 °C. The enzyme was found to be excessively heat stable, displaying a half-life of about 12 days and 5 days at 65 °C and 70 °C, respectively. L-RI_M_ catalysis conducted at slightly acidic pH of 6.0 and 70 °C achieved biosynthesis of about 30 g L^−1^ from 100 g L^−1^ d-allulose in 3 h.

**Key points:**

• *The present study explored an extreme temperature metagenome to identify a novel gene that encodes a thermostable l-rhamnose isomerase (L-RI*_*M*_*)*

• *L-RI*_*M*_ *exhibits substantial (80% or more) activity in a broad spectrum of pH (6.0 to 9.0) and temperature (70 to 80 °C) ranges*

• *L-RI*_*M*_ *is excessively heat stable, displaying a half-life of about 12 days and 5 days at 65 °C and 70 °C, respectively*

**Supplementary Information:**

The online version contains supplementary material available at 10.1007/s00253-024-13074-w.

## Introduction

Rare sugars include several monosaccharides and their derivatives, which sparsely exist in nature, e.g. d-allulose, trehalose, turanose, trehalulose, and d-allose. These sugars have been seeking attention for several years because of their multifarious applications in the food and pharmaceutical industries. These could be used as low-calorie sweeteners, inhibitors of microbial growth, bulking agents, cryoprotectants, etc. (Morimoto et al. 2006). d-Allose is a rare sugar with many beneficial properties to human health. This aldohexose is a C3 epimer of d-glucose. It is an ultra-low calorie sugar, retaining about 80% of the sweetness of sucrose (Xu et al. 2017). This monosaccharide was first isolated as a 6-*O*-cinnamyl glycoside from the leaves of an African shrub, *Protea rubropilosa* (Perold et al. 1973). A pharmacologically important Indian seaweed, *Halodule pinifolia*, contains about 3.7% d-allose (Kannan et al. 2012). The other plants that have been recorded to contain ultra-low amounts of d-allose are *Tamarindus indica*, *Acalypha hispida* leaves, and *Crataeva nurvala* (Chen et al. 2018b; Shintani et al. 2020). Its traces have also been detected in human cord blood (Hashimoto et al. 2013). The non-toxic property of d-allose has been established (Iga et al. 2010). The potential of d-allose has been demonstrated as a cryoprotectant (Sui et al. 2007), anti-oxidant (Nakamura et al. 2011), anti-microbial (Bautista et al. 2000), anti-inflammatory (Gao et al. 2013), tumour suppressive (Khajeh et al. 2023; Malm et al. 2015), and immuno-suppressive (Hossain et al. 2000; Takao et al. 2022) agent. Further, it can provide protection from ischemic reperfusion injury (Shinohara et al. 2016). Its role in plant growth metabolism or as an immunity regulator has also been studied. It may act as a triggering molecule against reactive oxygen species (ROS) (Kano et al. 2013; Mijailovic et al. 2021; Zhang et al. 2020). The monosaccharide is also considered a potential alternative to table sugar (sucrose) in the food industries (Zheng et al. 2022).

There is a chemical as well as a biological path for its synthesis. Its chemical synthesis is associated with drawbacks like low productivity, increased purification steps, and toxic by-products, which are environment-unfriendly (Choi et al. 2021). On the other hand, the biological approach involves enzymatic bioconversion of d-allulose into d-allose. The enzymes capable of catalyzing the conversion reaction between d-allulose and d-allose are l-rhamnose isomerase, d-ribose-5-phosphate isomerase, d-galactose-6-phosphate isomerase, mannose-6-phosphate isomerase, and glucose-6-phosphate isomerase (Chen et al. 2021). Among these, glucose-6-phosphate isomerase, mannose-6-phosphate isomerase, and d-galactose-6-phosphate isomerase exhibit low activity and by-product formation and therefore may not be useful for industrial applications (Chen et al. 2018b; Lim and Oh 2011). l-rhamnose isomerases and d-ribose-5-phosphate isomerases have been well characterized for d-allose production (Chen et al. 2018b; Turner et al. 2007). However, l-rhamnose isomerases have been shown to achieve the maximum d-allulose to d-allose conversion level of about 37% (Bai et al. 2015).

l-rhamnose isomerase (L-RI) catalyzes the reversible isomerization of d-allulose into d-allose. It has been reported from various bacterial sources, including *Escherichia coli*, *Bacillus pallidus*, *Bacillus subtilis, Bacillus halodurans*, *Thermotoga maritima*, *Caldicellulosiruptor saccharolyticus*, *Mesorhizobium loti*, *Dictyoglomus turgidum*, *Thermoanaerobacterium saccharolyticum*, and *Pseudomonas stutzeri* (Seo et al. 2018). These enzyme variants exhibit activity in the pH 7.0 to 9.0 range and temperature 60 to 90 °C. Heat-tolerant biocatalysts facilitate driving reactions at temperatures higher than ambient, which offers elevated reaction rates due to decreased viscosity, high diffusion rates, increased solubility, and lower risk of contamination issues (Turner et al. 2007). However, alkaline pH catalysis often results in a Maillard reaction and the generation of unwanted by-products. Therefore, a biocatalyst performing the low pH catalysis at moderate to high temperature is preferable for d-allose production. The present study describes the identification of a novel gene for l-rhamnose isomerase from a thermal aquatic habitat metagenome and its characterization for d-allose production with desirable catalytic attributes.

## Materials and method

### Materials

*Escherichia coli* Top 10 and BL21 (DE3) strains were purchased from Invitrogen (Waltham, MA, USA). The pET28a ( +) shuttle vector was procured from Novagen (Darmstadt, Germany). The enzyme Q5 DNA polymerase was obtained from Thermo Fisher Scientific (MA, USA). d-Allulose was purchased from Splenda, USA. Other chemicals and reagents used in the experiments were purchased from trustworthy suppliers and were of analytical grade, like Merck, TCI, HiMedia, CDH, and Bio-Rad.

### Gene mining and sequence analysis

The metagenomic sequence data generated from a hot aquatic body located in Tattapani geothermal field, Surguja, Chattisgarh, India (Kaushal et al. 2018), was mined for the identification of a novel gene encoding l-rhamnose isomerase. The sequence similarity analysis of the putative l-rhamnose isomerase gene (*rha*_M_) was performed by following the BLASTx algorithm against the NR Database of NCBI. A phylogenetic tree was constructed by the input of the peptide sequences of L-RI_M_ and homologous sequences in the MEGA X tool, following the neighbor-joining algorithm. To assess the evolutionary distance between diverging lineages, the bootstrapping test was conducted with 1000 iterations and following the Poisson-Correction method. To identify conserved residues and domains in the translated gene (*rha*_*M*_) sequence, i.e. L-RI_M_, multiple sequence alignment (MSA) was performed using the Clustal Omega, taking homologous protein sequences from the NCBI database.

The three-dimensional structure of L-RI_M_ was predicted using the AlphaFold2 (https://colab.research.google.com/github/sokrypton/ColabFold/blob/main/AlphaFold2.ipynb) and SWISS Model (https://swissmodel.expasy.org/) tools. The homology structure was visualized and superimposed on the best hit template using the UCSF Chimera (https://www.cgl.ucsf.edu/chimera) and PyMOL (https://pymol.org/). The Ramachandran plot analysis was done using UCSF Chimera and SWISS Model tools.

### In silico molecular docking

The docking studies were performed using AutoDock tools. The best possible conformations and binding affinities were selected on the basis of the default score list provided by the software. The interacting residues were within the range of < 5 Å. The results were visualized using Chimera and PyMol.2 molecular graphics system (https://pymol.org/2/).

### Circular dichroism (CD) analysis of L-RI_M_

A secondary structure composition study of L-RI_M_ was done by performing circular dichroism analysis in a Jasco J-1500 Circular Dichroism spectrophotometer (Jasco Corporation, Cremella (LC), Italy), equipped with a Xenon lamp, in the far-UV region of 190–260 nm. The protein (L-RI_M_) sample of a concentration of 8 µM, prepared in 50 mM potassium phosphate buffer (pH 7.0), was introduced in the CD spectrophotometer. The absorption was taken in a quartz cuvette with a path length of 2 mm and temperature 25 °C in a nitrogen-filled environment. Multiple scans (triplicate) were taken and averaged to prepare a final spectrum with a bandwidth of 2 nm and a step width of 0.1. The baseline spectra obtained by scanning buffer were subtracted from the spectrum obtained after each scan. The data obtained were further analyzed online through the CD analysis tool DichroWeb with CONTIN algorithm using a reference dataset SP175t, containing a large number of proteins with high-quality secondary structure data (http://dichroweb.cryst.bbk.ac.uk/).

### Gene cloning and protein expression

The putative *rha*_*M*_ gene was amplified from the hot spring metagenome DNA sample using Phusion High-Fidelity DNA Polymerase and gene-specific forward (F 5′ CGCGCGGCAGCCATATGATGACCTTTCCTGCTCCAAC3′) and reverse primers (R 5′- GGTGGTGGTGCTCGAGTCAGCGCGCAGCCAATAC3′). The amplified DNA was cloned in pET28a ( +) vector under the restriction sites, *Nde*I and *Xho*I, by using NEBuilder HiFi DNA Assembly master mix (NEB, Ipswich, USA) and following the Gibson assembly principle. The construct was introduced in the expression host, *E. coli* BL21, for gene expression and production of His-tagged L-RI_M_ protein. The recombinant cells were cultured in Luria–Bertani (LB) medium containing 10 mg L^−1^ kanamycin as a selection marker, then diluted in 200 mL LB with kanamycin at 37 °C/200 RPM to achieve a growth with 0.6 OD_600_. Gene expression was induced by 0.5 mM isopropylthio-β-galactoside (IPTG), followed by incubation of the cell culture at 16 °C/150 RPM for nearly 16–20 h. The culture was harvested by centrifugation at 4 °C/6000 RPM for 5 min. The cell pellet was washed with 0.85% saline water and re-suspended in lysis buffer (50 mM HEPES [pH7.0] and 300 mM NaCl). The cells were sonicated for 3 min (3 s pulse on and 10 s off) at an amplitude of 30 (Q Sonica, USA) to disrupt cells, releasing proteins into the buffer. The lysate was centrifuged at 4 °C/10,000 RPM for 45 min to pellet down the cellular debris. The crude cell extract was subjected to affinity chromatography in a nickel-nitrilotriacetic acid (Ni–NTA) matrix column (Qiagen) for separation and purification of His-tagged L-RI_M_ protein. The Ni–NTA column was equilibrated with the equilibrium buffer (50 mM HEPES buffer [pH 7.0], 300 mM NaCl, and 10 mM imidazole). The crude cell extract was then passed through a pre-equilibrated Ni–NTA column. The column was washed with wash buffer (50 mM HEPES buffer [pH 7.0], 300 mM NaCl, and 40 mM imidazole). Finally, His-tagged L-RI_M_ protein was eluted in 500 µL aliquots by using elution buffer (50 mM HEPES buffer [pH 7.0], 300 mM NaCl, and 300 mM imidazole). The protein purification was performed in AKTA pure protein purification system at 4 °C. This was followed by membrane dialysis to remove salt and imidazole. The concentrated protein fraction of L-RI_M_ was obtained by using an amicon filter (cut off 30 kDa, Merck). The protein concentration was determined by Bradford assay using the Bradford reagent (Sigma-Aldrich, St. Louis, Missouri) with bovine serum albumin (BSA) as a standard.

### Native and SDS-PAGE analyses

The protein samples were run in 12% Sodium Dodecyl Sulphate Polyacrylamide Gel Electrophoresis (SDS-PAGE) and 10% Native Polyacrylamide Gel Electrophoresis (Native PAGE) for molecular mass determination of the desired protein, L-RI_M_, in monomeric and native forms, respectively. For staining, the gel was immersed in 0.3% (w/v) Coomassie Brilliant Blue R-250, dissolved in a mixture of glacial acetic acid, methanol, and water in a ratio of 4.5:4.5:1, respectively. The gel was de-stained in a de-staining solution containing a mixture of glacial acetic acid, methanol, and water in the ratio of 0.75:1:8.25, respectively.

### Molecular mass determination

Size exclusion chromatography was performed to determine the molecular mass of L-RI_M_ protein. The standard proteins (Gel Filtration Calibration Kit, GE Healthcare) with a range of different molecular masses were determined. The sample and standards (Ovalbumin, MW 43 kDa; Conalbumin MW 75 kDa; Aldolase MW 158 kDa and Ferritin MW 440 kDa) were passed through Superdex 200 increase 10/300 GL (GE healthcare) using the mobile phase (50 mM Tris–Cl [pH 7.0] and 150 mM NaCl) at a flow rate of 0.5 mL.min^−1^ in AKTA pure (GE healthcare) system.

### Enzyme activity

The standard enzyme assays were conducted in 20 mM HEPES buffer (pH 7.0), containing 1 mM Mn^2+^ ions as a cofactor and 10 mM d-allulose as substrate, treated with 0.1 mg.mL^−1^ enzyme at 75 °C for 10 min. The reaction was terminated by boiling at 100 °C for 10 min. The activity of the enzyme was determined by measuring the amount of d-allose produced via catalytic transformation of d-allulose in HPLC (high-performance liquid chromatography).

### Effect of pH on enzyme’s activity and stability

To examine the effect of pH on enzyme’s activity, standard enzymatic reactions were performed in a wide pH range (pH 4.0 to 10.0) by using different buffers (20 mM), e.g. sodium acetate (pH 4.0 to 5.5), sodium phosphate (pH 6.0 to 6.5), HEPES (pH 7.0 to 8.5), and glycine–NaOH (pH 9.0 to 10.0). The pH stability was performed by incubating the enzyme (L-RI_M_) in the aforementioned buffers for 2 h at room temperature (25 °C), followed by conducting standard enzymatic reactions.

### Effect of temperatures on enzyme’s activity and stability

To determine the effect of different temperatures on enzyme activity, standard enzyme assays were performed over a wide temperature range (60 to 90 °C), followed by recording the relative activity of L-RI_M_. To examine heat stability, the aliquots of L-RI_M_ were exposed to heat (60 to 90 °C), and then standard enzyme assays were conducted at different time points to measure residual activity.

### Effect of metal ions on enzyme’s activity

Enzymatic reactions were conducted in the presence of 1 mM metal ions (Na^+^, K^+^, Li^+^, Fe^++^, Mn^++^, Cu^++^, Co^++^, Ni^++^, and Mg^++^) under standard assay conditions, and relative activity of L-RI_M_ was recorded. The reaction performed without metal was taken as a control.

### Enzyme kinetics

Standard enzyme assays were performed by taking variable substrate concentrations (1 to 400 mM d-allulose), and the kinetic parameters were measured by following the Michaelis–Menten kinetics and the Lineweaver–Burk plot analysis.

### Substrate specificity

Enzyme assays were conducted with a variety of substrates, e.g. d-allulose, d-allose, l-rhamnose, d-ribose, d-galactose, and d-glucose, under standard reaction conditions, and relative activity was recorded.

### d-Allose production

d-Allulose (100, 200, and 400 g L^−1^) was treated with L-RI_M_ (0.1 mg/mL) at slightly acidic pH (HEPES pH 6.0) at 70 °C for 6 h. The reaction product was analyzed in HPLC after each hour of reaction.

### HPLC analysis

Reaction samples, passed through a 0.22-µm filter, were prepared in 50% acetonitrile and analyzed in HPLC (1260 Infinity, Agilent Technologies) equipped with Zorbax NH_2_ column (4.6 × 250 mm, 5 µm ID, Agilent Technologies). The column and refractive index detector (RID) temperatures were 40 °C and 45 °C, respectively. Acetonitrile (63% v/v) mixed in degassed Milli-Q water was used as the mobile phase, with a flow rate of 1 mL.min^−1^.

### Statistical analysis

All the experiments were performed in triplicates with three independent experiments, and the data is represented as the average of the data along with the standard deviation. The statistical analysis of the variance of data has been performed by one-way ANOVA (version 14.0) and Minitab 21 software.

## Results

### Sequence analysis and 3D homology structure

**Fig. 1:**
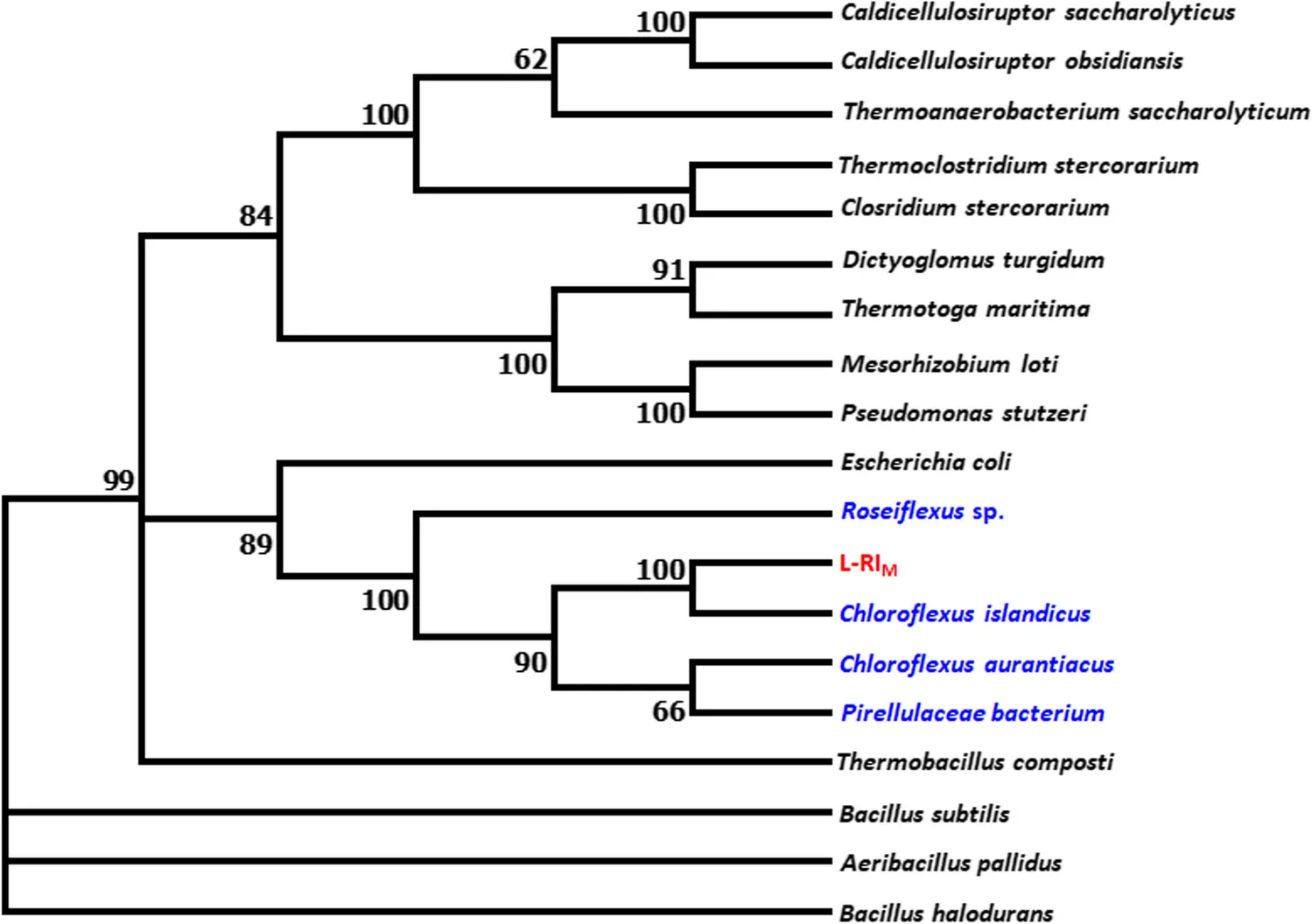
The phylogenetic relationship among L-RI_M_ and homologous l-rhamnose isomerase (peptide) sequences. The source and accession numbers of the sequences are as follows: *Caldicellulosiruptor saccharolyticus* (ABP66492), *Caldicellulosiruptor obsidiansis* (ADL41970), *Thermoanaerobacterium saccharolyticum* (ADF43732), *Thermoclostridium stercorarium* (AGC67668), *Closridium stercorarium* (AGC67668.1), *Dictyoglomus turgidum* (WP_012582814.1), *Thermotoga maritima* (AGL49999), *Mesorhizobium loti* (WP_109666615), *Pseudomonas stutzeri* (BAD14073), *Escherichia coli* (KXG97253.1), *Roseiflexus* sp. (GIW02602.1), *Chloroflexus islandicus* (WP_066785062), *Chloroflexus aurantiacus* (WP_012258147.1), *Pirellulaceae bacterium* (GIW91192.1), *Thermobacillus composti* (AGA57429.1), *Bacillus subtilis* (ARW32823.1), *Aeribacillus pallidus* (BAF80456.1), *Bacillus halodurans* (BAB05271.1), and L-RI_M_ (this study). The homologous sequences shown in blue colour in the figure are uncharacterized homologous sequences taken from NCBI database

**Fig. 2:**
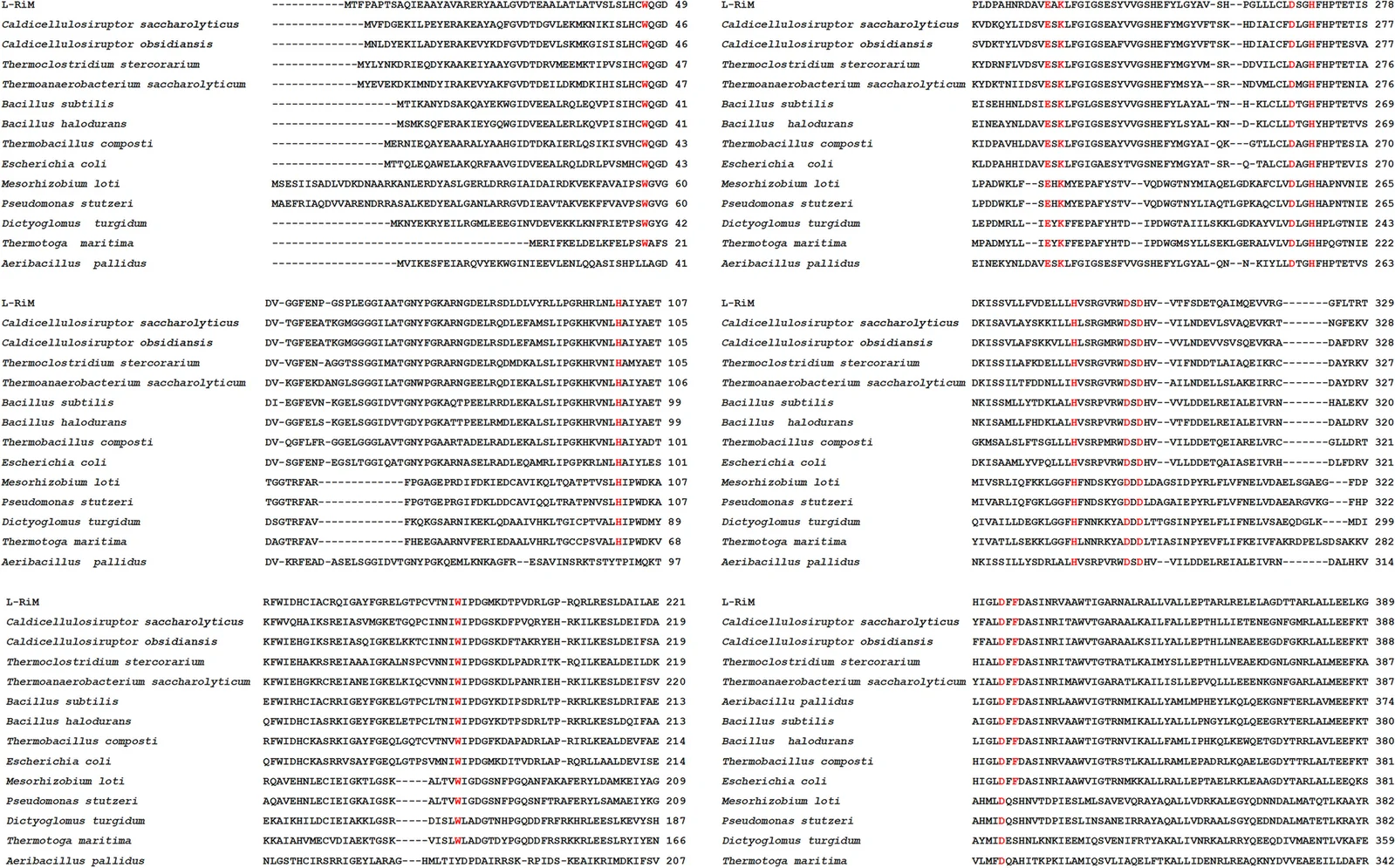
Multiple sequence alignment of L-RI_M_ with previously characterized l-rhamnose isomerases. Amino acids highlighted in red are conserved residues

**Fig. 3:**
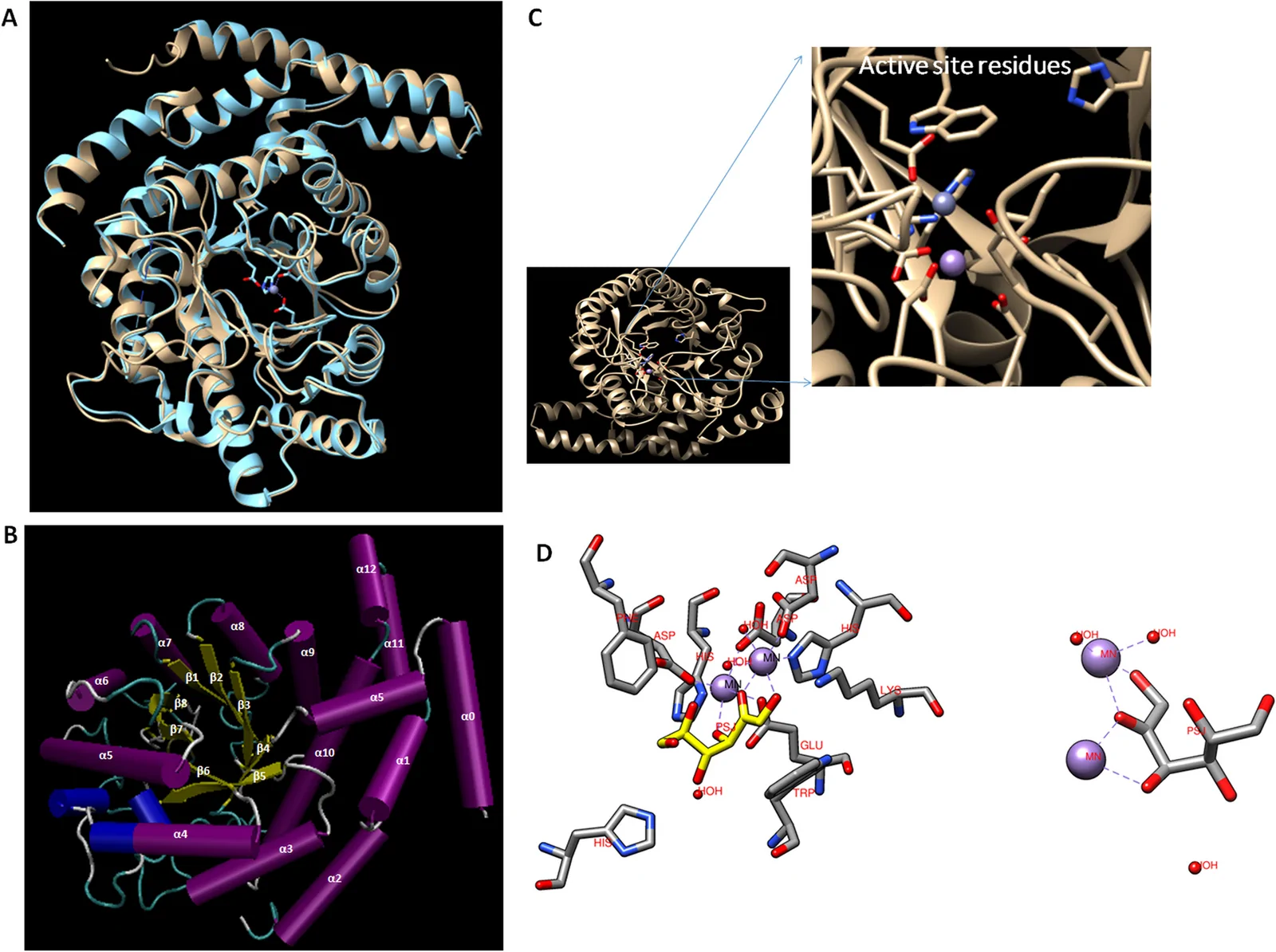
**A** Superimposed image of L-RI_M_ over the template, i.e. 3D structure of L-RI (blue) from *E. coli* K 12 (brown). **B** Three-dimensional homology structure of L-RI_M_ showing (α/β)8 barrel structure with additional α-helices and loops. **C** The catalytic site of L-RI_M_ and active site residues. **D** Critical amino acid residues involved in substrate and metal binding

A putative gene (*rha*_M_) encoding a peptide (L-RI_M_) representing l-rhamnose isomerase enzyme was identified and cloned from a hot aquatic habitat metagenome (Kaushal et al. 2018). BLASTn analysis of the metagenomic sequence (*rha*_M_) against the public NR database depicted about 79% identity with an unidentified sequence from *Chroroflexus aurantiacus*. At the protein level, L-RI_M_ was found phylogenetically close to an uncharacterized peptide sequence from *Chloroflexus islandicus*, exhibiting about 97% identity (Fig. 1). When compared with the characterized l-rhamnose isomerases, L-RI_M_ showed the maximum identity of 64.59% with l-rhamnose isomerase from *Escherichia coli* (KXG97253.1) and the minimum identity of 19% with *Pseudomonas stutzeri* and *Mesorhizobium loti*. Nevertheless, *E. coli* L-RI has not been characterized for d-allose biosynthesis. Multiple sequence alignment analysis with homologous peptide sequences revealed conserved residues in L-RI_M_ (Fig. 2). The conserved catalytic residues, his101 and asp334, are the possible critical residues to interact with O4 and O5 of d-allulose (Wu et al. 2009). Other conserved residues include his270, glu233, lys235, asp302, asp267, trp193, phe143, his294, asp304, phe336, and trp48 (Fig. 3C). Among these, glu233, asp267, his294, and asp334 could be involved in metal binding, which is vital for catalytic function (Chen et al. 2018a) (Fig. 3D).

**Fig. 4:**
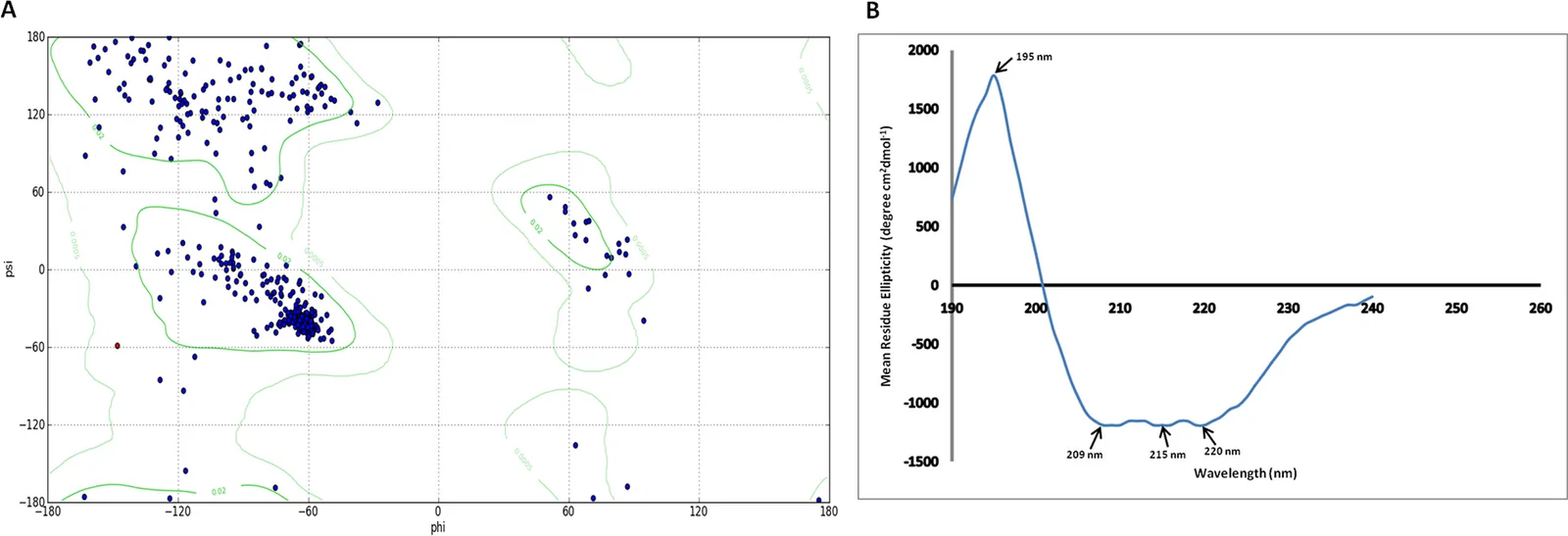
**A** Ramachandran plot showing occurrence of amino acid residues of L-RI_M_ homology structure in the allowed regions. **B** Far-UV circular dichroism spectra of L-RI_M_ at room temperature

The peptide sequence of L-RI_M_ was successfully modelled on the three-dimensional crystal structure of the template, *E. coli* K 12 (PDB ID: 1d8w), a homo-tetramer protein that belongs to the rhamnose isomerase family (divalent-metal-dependent TIM barrel enzymes). The superimposition of L-RI_M_ homology 3D structure onto the template revealed the RMSD value of 0.624 Å (Fig. 3A). The homology structure of L-RI_M_ depicted (β/α)_8_-barrel conformation with some additional α-helices (α0 and α9 to12) and the flexible loops (Fig. 3B). The Ramachandran plot analysis of the L-RI_M_ homology structure propounded the occurrence of about 95% amino acids in the Ramachandran favoured regions, with less than 1% Ramachandran outliers, witnessing appropriate folding incidences in the predicted protein model (Fig. 4A). The results advocated towards possible stability of the predicted structure of L-RI_M_.

### In silico molecular docking

l-rhamnose isomerase can accommodate monosaccharides, e.g. d-allulose, d-allose, and l-rhamnose, as the substrate for catalysis. In the present study, the homology structure of L-RI_M_, superimposed onto *E. coli* strain K12 l-rhamnose isomerase (PDB ID: 1d8w), was used for in silico molecular docking simulation studies. d-Allulose was docked against the active pocket of L-RI_M_. The docking studies revealed that oxygen 1, 2, and 3 of d-allulose interact with the metal ions 1 and 2 (Fig. 3D). The torsion angle of C4-C5-C6-O6 of d-allulose was computed to be − 66.6°. A hydrophobic pocket around O6 of d-allulose was formed by phenyl-alanine and tryptophan residues of l-rhamnose isomerase. The presence of the hydroxyl group at the C6 position of the substrate, like d-allulose, creates an unstable environment, leading to lower substrate affinity. On the other hand, L-RIs generally prefer the hydrophobic methyl group at the C6 position, which leads to a relatively higher affinity towards l-rhamnose.

### Circular dichroism (CD) analysis

The secondary structure analysis of L-RI_M_ by circular dichroism showed a positive peak at 195 nm and negative peaks at 209 nm, 215 nm, and 220 nm, with a crossover at 203 nm. These peaks correspond to a structure containing α-helices and β-strands (Greenfield 2006; Sreerama et al. 1999; Whitmore and Wallace 2008). The DichroWeb tool algorithm computed the proportions of α-helices, β-strands, turns, and unordered secondary structures in L-RI_M_ as 7.7%, 39.5%, 12.6%, and 40.2%, respectively. The NRMSD value of the experimental spectrum with the calculated reference set is 0.221 (Fig. 4B). The ‘turns’ are comprised of beta turns, bends, and bridges. The unordered structures, which include all the structures other than beta turns, bends, and bridges, had a significant proportion of the protein.

### Heterologous expression and purification

The heterologous gene expression was executed in *E. coli* by introducing different concentrations of the inducer (IPTG), i.e. 0.2 mM, 0.4 mM, 0.6 mM, 0.8 mM, and 1 mM, in the culture incubated at 16 °C. The culture was incubated at different temperatures, i.e. 37 °C, 30 °C, 25 °C, 20 °C, and 16 °C using 1 mM of the expression inducer (Fig. S1). The IPTG concentration of 1 mM and the temperature 16 °C were experienced to be favourable for a higher expression of recombinant protein. L-RI_M_ protein was extracted by cell lysis and then purified via affinity chromatography.

### Molecular mass determination

**Fig. 5:**
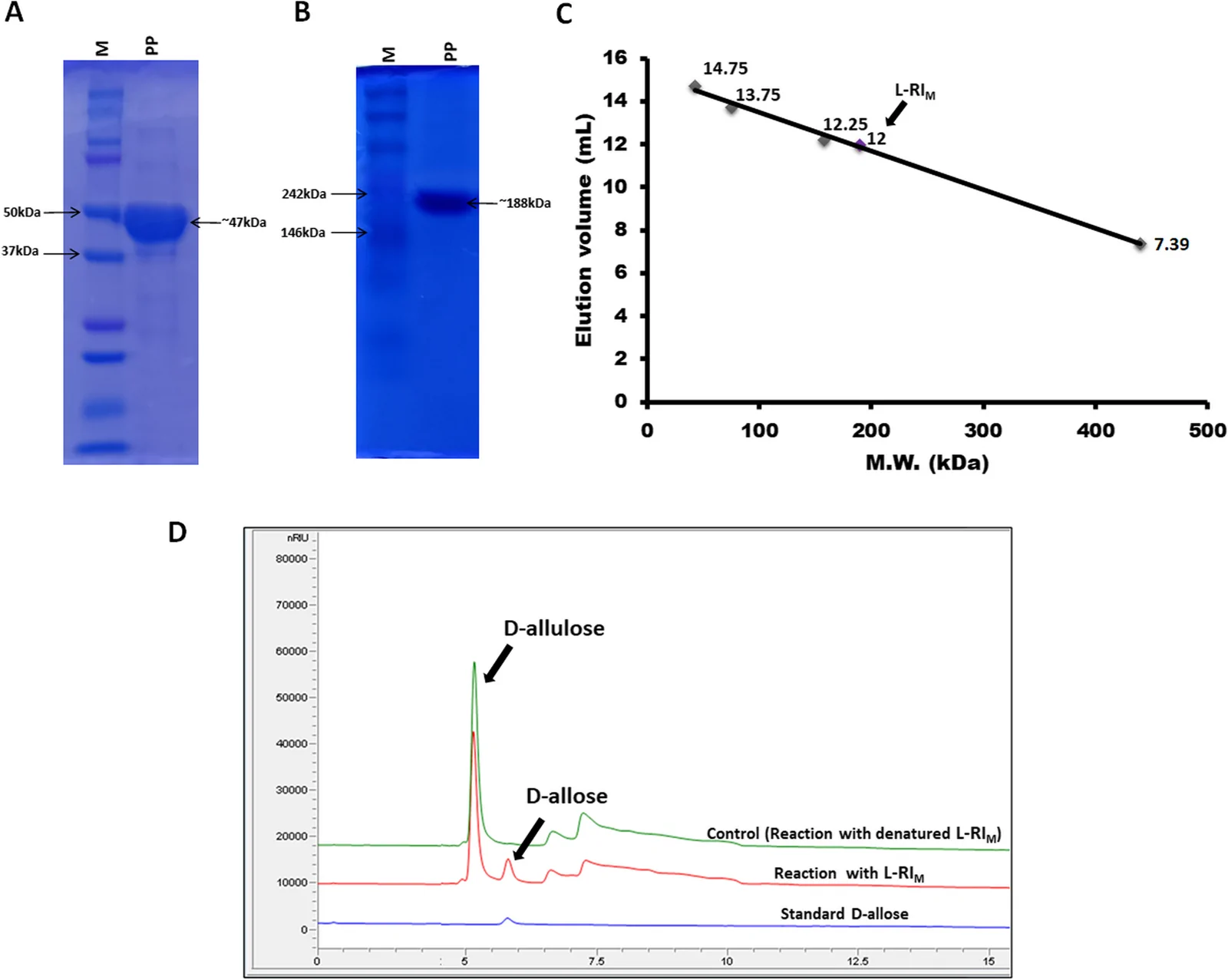
Molecular mass and catalytic function determination of L-RI_M_. **A** SDS-PAGE showing band of L-RI_M_ protein subunits. **B** Native-PAGE showing band of tetrameric L-RI_M_ protein (M, marker; PP, purified protein). **C** Size exclusion chromatogram of L-RI_M_ for native mass determination. **D** HPLC analysis of catalytic reaction product

The purified protein was then loaded on native and SDS-PAGE, which indicated more than 95% purity of the protein (L-RI_M_). The molecular mass of L-RI_M_ subunits was estimated to be ~ 47 kDa. Native–PAGE analysis showed a single band of ~ 190 kDa, divulging the native state of L-RI_M_ to be a homo-tetramer (Fig. 5A). The molecular mass of protein was further confirmed by size exclusion chromatography (Fig. 5B). The yield of L-RI_M_ was approximated to be about 70 g obtained from per litre of the recombinant *E. coli* culture. The standard enzyme assay with d-allulose resulted the production of d-allose (Fig. 5D), validating the catalytic function of L-RI_M_ as a variant of l-rhamnose isomerase enzyme.

### Effect of pH on L-RI_M_’s activity and stability

**Fig. 6:**
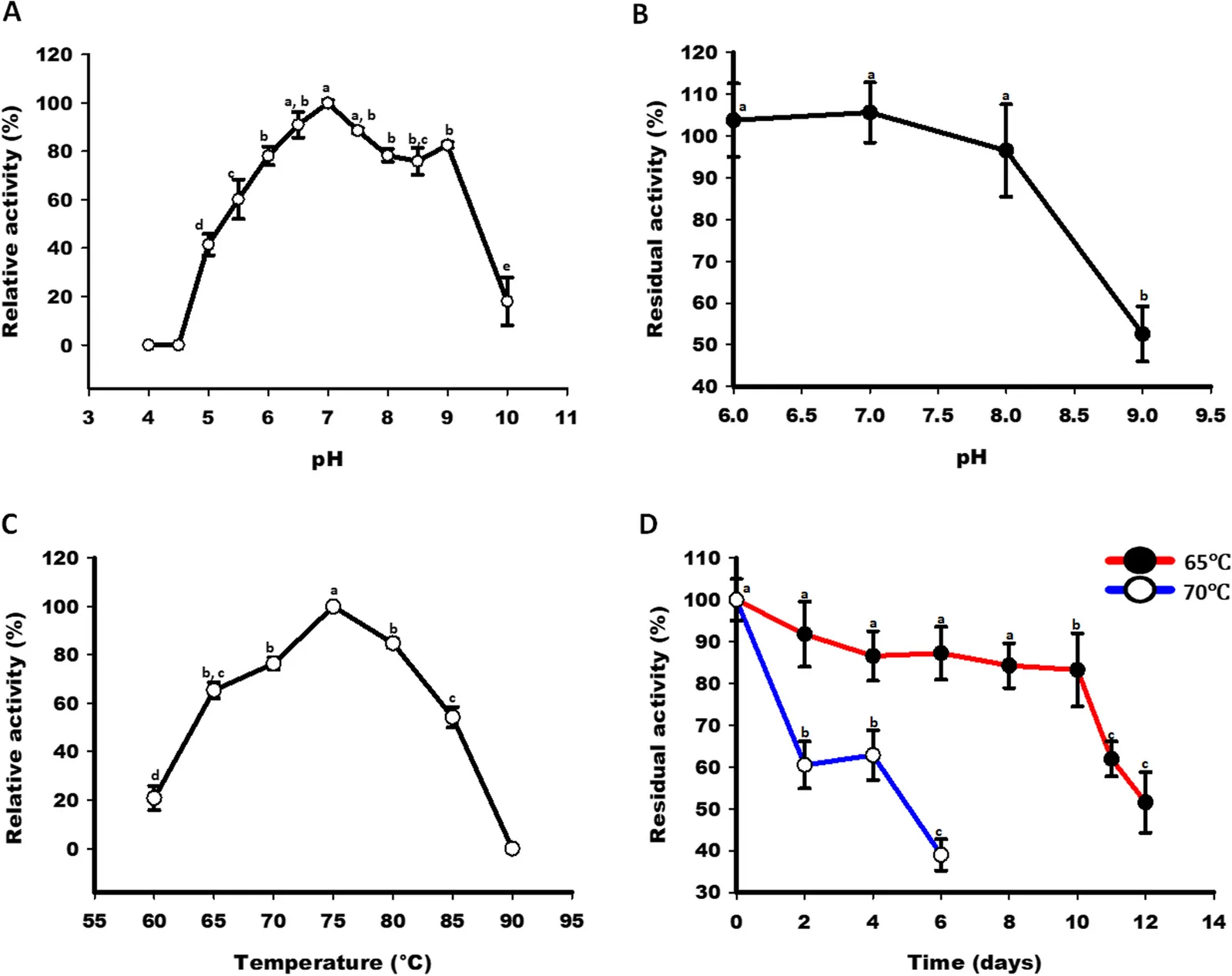
Effect of pH and temperature on the activity and stability of L-RI_M_. **A** Relative activity profile of L-RI_M_ at different pH. **B** Residual activity profile of L-RI_M_ after exposure to different pH. **C** Relative activity profile of L-RI_M_ at different temperatures. **D** Residual activity profile of L-RI_M_ after exposure to different temperatures. The mean values not sharing common alphabets show statistical differences at *P*-value < 0.05

The catalytic activity of L-RI_M_ was determined in buffers ranging from acidic to alkaline (pH 4.0 to 10.0). The enzyme was found functional in a broad range of pH, i.e. from 5.5 to 9.0, displaying 60 to 80% relative activity (Fig. 6A). The neutral pH was noted to be optimum for L-RI_M_ to transform d-allulose to d-allose. Nevertheless, catalytic reaction at a slightly acidic pH (6.0) could achieve 80% relative activity of L-RI_M_. The exposure of pH 6.0 to 7.0 for 2 h at room temperature did not lead to any significant loss to the stability of L-RI_M_. At pH 8.0, it retained more than 80% of residual activity after 2 h of incubation (Fig. 6B). The exposure to the alkaline pH of 9.0 was detrimental to the stability of L-RI_M_.

### Effect of temperature on L-RI_M_’s activity and stability

The activity profile of the L-RI_M_ enzyme was examined at different temperatures, from 60 to 90 °C. The enzyme manifested substantial activity (> 60%) at the high-temperature range of 65 °C to 85 °C (Fig. 6C). The optimum activity was obtained at 75 °C. L-RI_M_ was found to be a thermo-stable enzyme, retaining more than 80% residual activity even after heat (65 °C) exposure for 10 days. At the higher temperature (70 °C), it could retain 60% residual activity after 2 days of heat treatment (Fig. 6D).

### Influence of metals on L-RI_M_’s activity

**Fig. 7:**
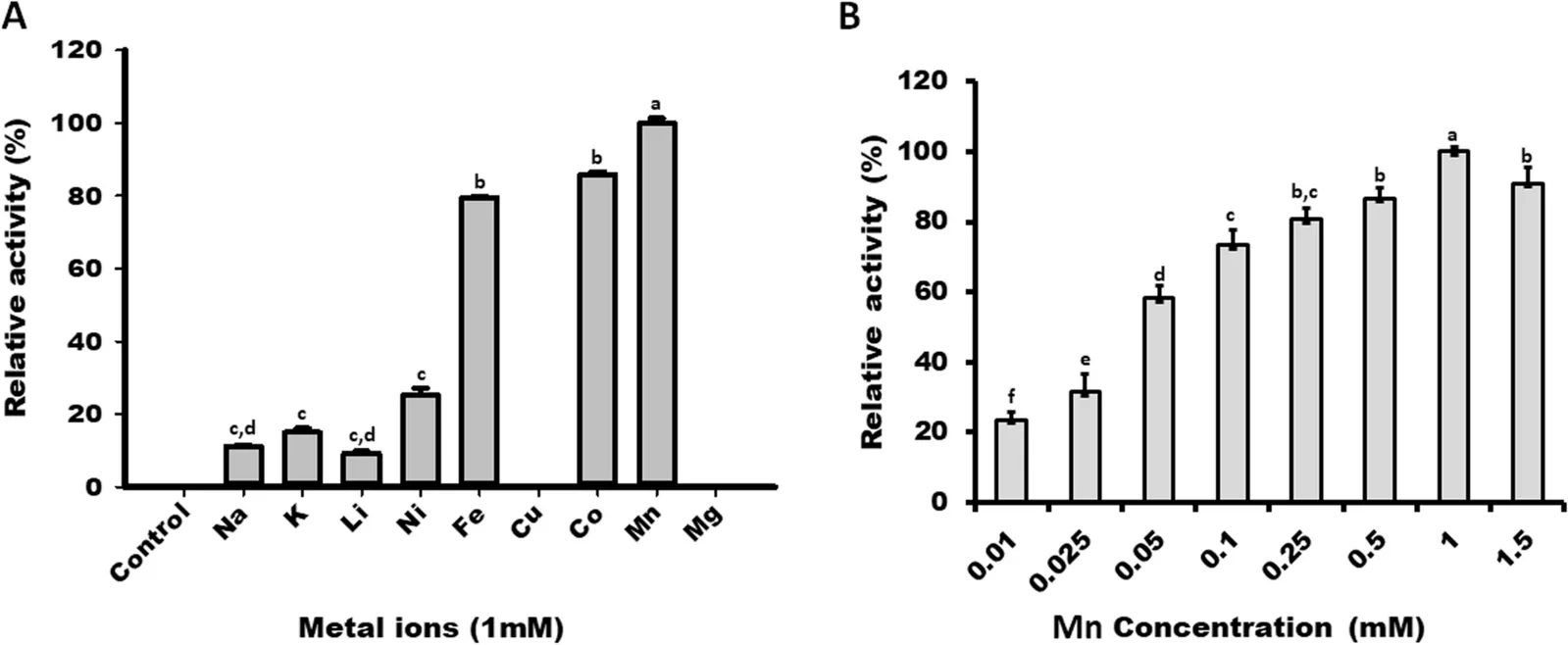
Effect of metal ions on the activity of L-RI_M_. **A** Relative activity profile of L-RI_M_ in the presence of 1 mM of different metals. **B** Relative activity profile of L-RI_M_ in the presence of different Mn concentration. The mean values not sharing common alphabets show statistical differences at *P*-value < 0.05

The enzyme assays were carried out by supplying different metal ions in the reaction. L-RI_M_ exhibited the maximum activity in the presence of Mn followed by Co (85%) and Fe (80%). Notably, the presence of Cu or Mg in the reaction completely abolished the catalytic function of L-RI_M_. The presence of Na, K, Li, and Ni was also shown to be detrimental to L-RI_M_’s activity. Interestingly, nil activity was noted in the case of catalytic reactions conducted without metal (Fig. 7A). Thus, Mn is critical to maintaining the structural integrity of the L-RI_M_. The concentration of 1 mM Mn was found sufficient to achieve the optimum activity of L-RI_M_ (Fig. 7B).

### Kinetics of the enzyme

The kinetic parameters of L-RI_M_ were computed towards the substrate, d-allulose, by following the Michaelis–Menten equation and Lineweaver–Burk plot analysis. The substrate binding affinity (*K*_m_), turnover number (*k*_cat_), and catalytic efficiency (*k*_cat_/*K*_m_) were calculated as 110 mM, 328.8 s^−1^, and 2.981 mM^−1^ s^−1^, respectively (Fig. S2).

### Enzyme activity towards different substrates

The catalytic activity of L-RI_M_ was determined towards different substrates, e.g. d-allulose, l-rhamnose, d-allose, d-ribose, d-galactose, and d-glucose. The maximum activity was recorded for l-rhamnose, followed by d-allulose, d-galactose, d-allose, d-glucose, and d-ribose (Fig. S3).

### Catalytic bioconversion of d-allulose to d-allose

L-RI_M_-catalyzed bioconversion was conducted under optimal reaction conditions for 10 to 160 min, taking 10 mM d-allulose concentration. The reversible enzymatic reaction resulted in the maximum transformation of about 40% d-allulose to d-allose in 120 min of enzymatic reaction (Fig. S4). In the case of bioconversion reaction conducted with a higher concentration of d-allulose, e.g. 100 g L^−1^, about 30 g L^−1^ d-allose could be yielded. Further increase in substrate concentration caused depletion in d-allose conversion yield percentage (Fig. S5).

## Discussion

l-Rhamnose isomerase is a promising biocatalyst for the bio-production of d-allose. d-Allose production was demonstrated by employing l-rhamnose isomerase from *Pseudomonas stutzeri* to establish a continuous bioreactor system (Morimoto et al. 2006). However, it leads to the generation of by-products like d-altrose, which is undesirable and hinders the separation of d-allose from d-allulose (Leang et al. 2004; Morimoto et al. 2006). l-rhamnose isomerases have been characterized from ten or more bacterial sources. However, poor thermostability, poor catalytic efficiency, and neutral to alkaline pH activity are significant limitations in the pilot-scale production of these high-value, rare sugar molecules (Patel et al. 2020). Therefore, extreme habitats need to be explored to identify novel variants of l-rhamnose isomerase, possibly equipped with higher stability and slightly acidic activity to produce d-allose with negligible or nil by-product formation. The extreme temperatures of hot water habitats are potential bio-resource of the arsenal of enzyme variants with higher stability and better kinetic attributes of industrial significance (Agarwal et al. 2019; Kaushal et al. 2018; Patel et al. 2020). In the present study, extreme temperature aquatic habitat metagenome mining was conducted, unearthing a novel gene encoding l-rhamnose isomerase (L-RI_M_) of *Chloroflexus* sp. origin. To the best of our knowledge, L-RI belonging to *Chloroflexus* sp. has not yet been characterized.

The amino acid sequence analysis suggested L-RI_M_ to be a member of a group that includes L-RIs characterized from *E. coli*, *Aeribacillus pallidus*, *B. subtilis*, and *B. halodurans*. The comparison of L-RI_M_ with homologous sequences divulged the presence of conserved residues for substrate binding and metal binding, which are critical in catalyzing the isomerization function via the metal-mediated hydride-shift mechanism (Xu et al. 2017, 2016). The homology model of L-RI_M_ depicted the (β/α) _8_-barrel conformation, which is in accordance with the stable and resolved structures of l-rhamnose isomerases. The extra α-helices (α0 and α9 to 12) and the flexible loops in L-RI_M_ homology structure could be critical in substrate recognition and catalytic function. Further, these additional α-helical domains are possibly involved in multi-merization of the protein (KorndoÈrfer et al. 2000). The in silico molecular docking study provided insights into the interaction of d-allulose and the active site residues of L-RI_M_, endorsing the hydride shift mechanism of d-allulose to d-allose isomerization. Thus, the sequence analysis of L-RI_M_ and molecular docking studies gave sufficient confidence to proceed with the heterologous expression of the metagenomic DNA fragment.

The circular dichroism analysis of L-RI_M_ revealed the presence of α-helices and β-strands in the protein, strengthening the homology structure prediction (Fig. 3). However, the higher NRMSD value of 0.221 (which should be > 0.1) indicated poor correspondence between experimental and the best-fit spectra. This could be a reason for the detection of a relatively lower proportion of α-helices, with more β-strands in L-RI_M_ protein, as compared to the homology model (Miles et al. 2021). Otherwise, the CD results follow the predicted secondary structure.

The molecular mass analysis of the protein (L-RI_M_) subunits in SDS-PAGE stipulated a ~ 47 kDa band, equal to the amino acids' cumulative mass. The native-PAGE and size exclusion chromatography results suggested a tetrameric configuration of L-RI_M_. Most of the characterized l-rhamnose isomerases are reported to be tight homo-tetramer of four (β/α)_8_-barrels, except enzymes from *Bacillus halodurans*, which is a dimer and a trimer from *Clostridium stercorarium* (Prabhu et al. 2011; Seo et al. 2018)*.*

Most of the l-rhamnose isomerases characterized so far exhibit the maximum range of d-allulose to d-allose isomerization activity at alkaline or neutral pH (Seo et al. 2018). However, the flexible pilot production of d-allose demands the enzyme’s catalytic function in a broad pH spectrum. L-RI_M_, being active in the range of 5.0 to 9.0 pH, is suitable for industrial use. Its noteworthy activity (60–90%) at slightly acidic pH (5.5 to 6.5) is desirable to avoid browning and by-product formation due to the Mailliard reaction during catalysis (Patel et al. 2020). On a similar note, L-RIs from *Thermobacillus composti* KWC4 and *C. obsidiansis* have been demonstrated to display substantial activity (60%) at slightly acidic pH (Chen et al. 2018c; Xu et al. 2017); however, L-RI_M_ in this study showed relatively superior active site functioning in terms of executing substrate’s conversion (i.e. d-allulose to d-allose) with a higher turnover number. Its *k*_cat_ was recorded to be the second highest (after that of *Psedomonas stutzeri*) among the characterized L-RIs.

High-temperature bio-catalysis favours sugar biotransformation reactions by avoiding contamination, increasing substrate solubility, and achieving a faster reaction rate (Xu et al. 2016). The high-temperature activity of L-RI_M_ in the range of 65–85 °C was consistent with most of the L-RIs characterized from different bacterial sources, the temperature effect analysis of which has been reported to exhibit activity at 60 °C or more. However, remarkable variations in thermal stability have been noted among L-RI variants (Table 1). Thermal stability is an important intrinsic property of the enzyme that critically impacts the feasibility of the industrial bioprocess (Nezhad et al. 2022). The ability to tolerate high temperature surroundings (65–70 °C), and exhibiting the half-life of several (4 to 12) days, endorses L-RI_M_ to be a potential biocatalyst for pilot scale d-allose production. The composition of amino acid residues in an enzyme is critical for its behaviour in different environmental conditions. The presence of charged amino acids at the interacting surfaces of multimeric protein (Fig. S6) is considered crucial for its high thermal stability (Holden 2009). In addition, the presence of a large number of hydrophobic residues (Fig. S6) also contributes to the protein’s thermal stability by providing a robust hydrophobic core (Holden 2009). Notably, L-RI_M_ exhibited the highest thermostability among characterized L-RIs after *Thermotoga maritima* (Table 1). However, the heat exposure in the absence of manganese was detrimental to L-RI_M_, suggesting metal is a critical cofactor for executing catalysis and maintaining the structural integrity of L-RI_M_ protein. The metal-dependent activity and stability were in accordance with the previous reports on other L-RIs (Chen et al. 2018b; Turner et al. 2007). The homology model of L-RI_M_ depicted two metal binding sites. One metal ion possibly accomplishes bonding between substrate and protein to achieve conformational stability, and the other metal ion subsequently facilitates isomerization activity via the hydride-shift mechanism (Yoshida et al. 2010, 2013). Further, the hydrophobic residues near the substrate binding site’s fourth, fifth, and sixth positions could be responsible for the limited substrate specificity of the protein (Yoshida et al. 2013).

**Table 1:** Comparison of physicochemical properties of L-RI_M_ and previously characterized l-rhamnose isomerases for d-allose biosynthesis

Enzyme source	Optimum pH	Optimum temperature (°C)	Cofactor	Half-life (hours)	*K*_m_ (mM)	Turnover number *k*_cat_ (s^−1^)	*k*_cat_/*K*_m_ (mM^−1^ s^−1^)	Equilibrium (d-allulose to d-allose) (%)	References
*Pseudomonas stutzeri* (BAD14073)	9	60	Mn	0.1 (50 °C)	42	2500	59.5	25	Leang et al. 2004
*Bacillus pallidus* Y25 (BAF80456.1)	7	65	Mn, Co	1 (60 °C)	41.8	34.5	0.825	35	Sui et al. 2007
*Thermoanaerobacterium saccharolyticum* (ADF43732)	7	75	Co, Mn	2 (70 °C)	121	33.9	0.28	34	Lin et al. 2010
*Thermotoga maritima* (AGL49999)	8	85	Mn	773 (75 °C)	NR	NR	NR	NR	Park et al. 2010
*Mesorhizobium loti* (WP109666615)	9	60	Mn, Co	> 1 (50 °C)	NR	1.33	NR	NR	Takata et al. 2011
*Caldicellulosiruptor saccharolyticus* ATCC 43494 (ABP66492)	7	90	Mn	6 (80 °C)	14.3	68.1	4.77	33	Lin et al. 2011
*Dictyoglomus turgidum* (WP012582814.1)	8	75	Mn	71.3 (65 °C)	61.5	81	1.3	NR	Kim et al. 2013
*Bacillus subtilis* 168 (ARW32823.1)	8.5	70	Mn	10 (60 °C)	5.98	0.74	0.12	37	Bai et al. 2015
*Thermobacillus composti* (AGA57429.1)	7.5	65	Mn	10 (60 °C)	70.4	2.46	0.035	23.34	Xu et al. 2017
*Caldicellulosiruptor obsidiansis* OB47 (ADL41970)	7	90	Co, Mn	1 (90 °C)	25.8	11.25	0.44	25	Chen et al. 2018c
*Clostridium stercorarium* (AGI38715.1)	7	75	Mn	22.8 (65 °C)	17.2	36.3	2.11	33	Seo et al. 2018
*Bacillus subtilis* (AL009126)	9	70	Mg	NR	NR	NR	NR	NR	Li et al. 2020
L-RI_M_ (OR296619)	7.5	75	Mn	288 (65 °C)	110.3	328.8	2.981	40	This study

The increase in substrate concentration (e.g. from 100 mM to 100 g L^−1^) negatively affected the catalytic conversion of d-allulose to d-allose. This could be because a higher substrate concentration causes increased viscosity of the reaction mixture, which interferes with the substrate’s access to the catalytic domain of the enzyme (Liu 2020). A higher viscosity of the surrounding possibly exerts a negative impact on the structural integrity of the enzyme, which in turn affects the catalytic efficiency of the enzyme (Uribe and Sampedro 2003).

In conclusion, the present investigation reports a gene derived from a hot-spring metagenome that encodes for l-rhamnose isomerase (L-RI_M_). This protein of *Chloroflexus* sp. origin catalyzes the reversible bioconversion of d-allulose to d-allose. The detailed enzyme characterization experiments divulged the catalytic capability of L-RI_M_ to produce d-allose in slightly acidic to alkaline pH and at high-temperature conditions. Its excessive heat stability and reasonably good turnover number postulated L-RI_M_ as a suitable biocatalyst for pilot-scale production of d-allose, a rare sugar of high importance to the pharmaceutical industry. However, l-rhmanose isomerase–based bioprocess is limited by using a costly substrate, d-allulose. An enzymatic process conducted with a relatively low-cost substrate, e.g. d-fructose and d-glucose, could be a more economical approach for the industrial production of d-allose.

## Supplementary Information

Below is the link to the electronic supplementary material.

Supplementary file1 (PDF 694 KB)

## Data Availability

The sequence data associated with L-RI_M_ is available under the NCBI GenBank accession number OR296619.
