# Supplementary material for: A novel thermotolerant l-rhamnose isomerase variant for biocatalytic conversion of d-allulose to d-allose
Source: Appl Microbiol Biotechnol. 2024 Apr 2;108(1):279. doi: 10.1007/s00253-024-13074-w (PMC10987364; doi:10.1007/s00253-024-13074-w)
Supplement: Supplementary file 1 — Supplementary file1 (PDF 694 KB) [file 253_2024_13074_MOESM1_ESM.pdf]

A novel thermotolerant L-rhamnose isomerase variant for biocatalytic conversion of D-allulose to D-allose

Sweety Sharma[1,2], Satya Narayan Patel[1], Sudhir P. Singh[1,*]

[1]Center of Innovative and Applied Bioprocessing, A national institute under the Department of Biotechnology (DBT), Govt. of India, NABI Campus, Sector 81, SAS Nagar, Mohali, India-140306

[2]Indian Institute of Science Education and Research Mohali, Sector 81, SAS Nagar, Mohali, India-140306

*Correspondence:
Sudhir P. Singh
Email: sudhirsingh@ciab.res.in
Tel: +91 172 5221415
Fax: +91 172 5221499

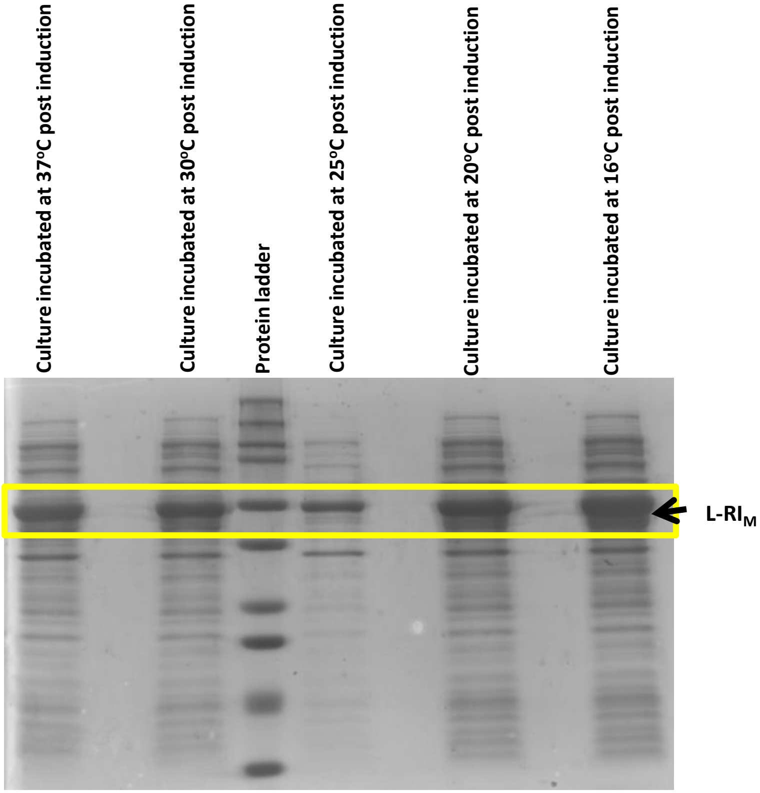

Fig. S1. Expression of L-RI$_M$ at different temperatures (37°C, 30°C, 25°C, 20°C, and 16°C) after IPTG induction.

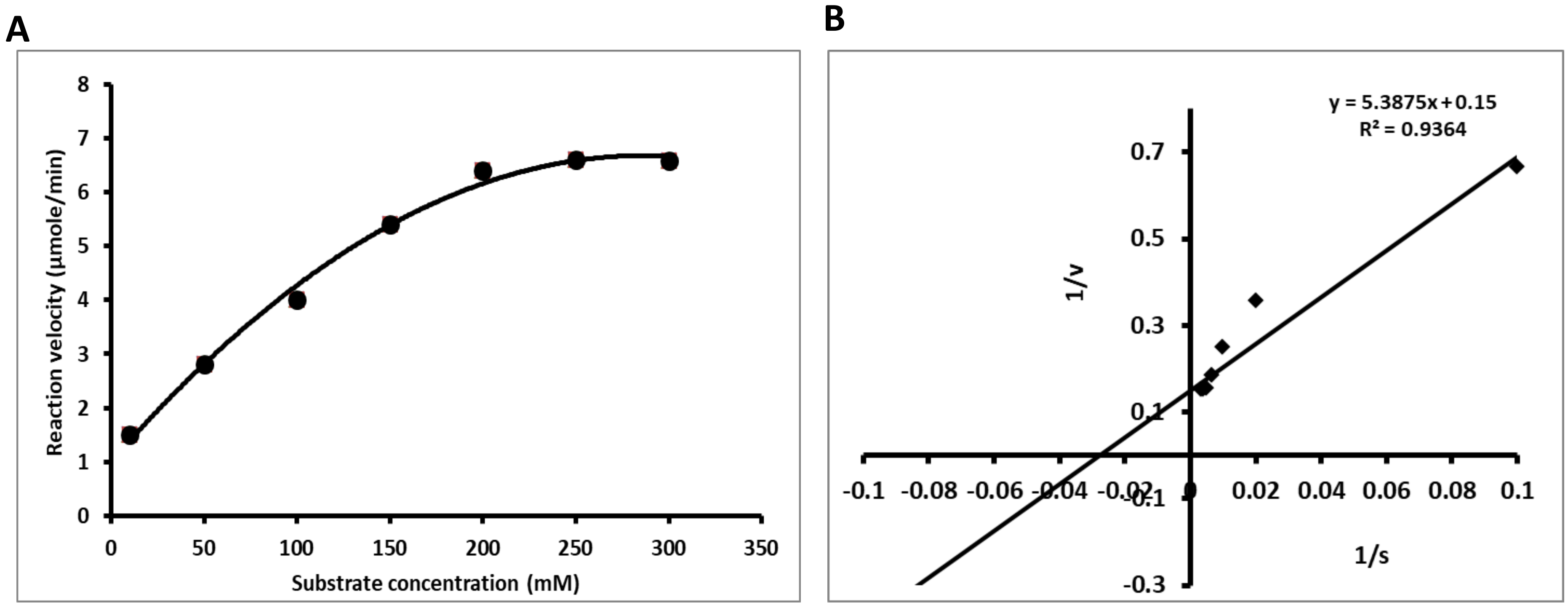

Fig. S2. A. Michelis-Menten Kinetic plot. **B.** Lineweaver-Burk plot

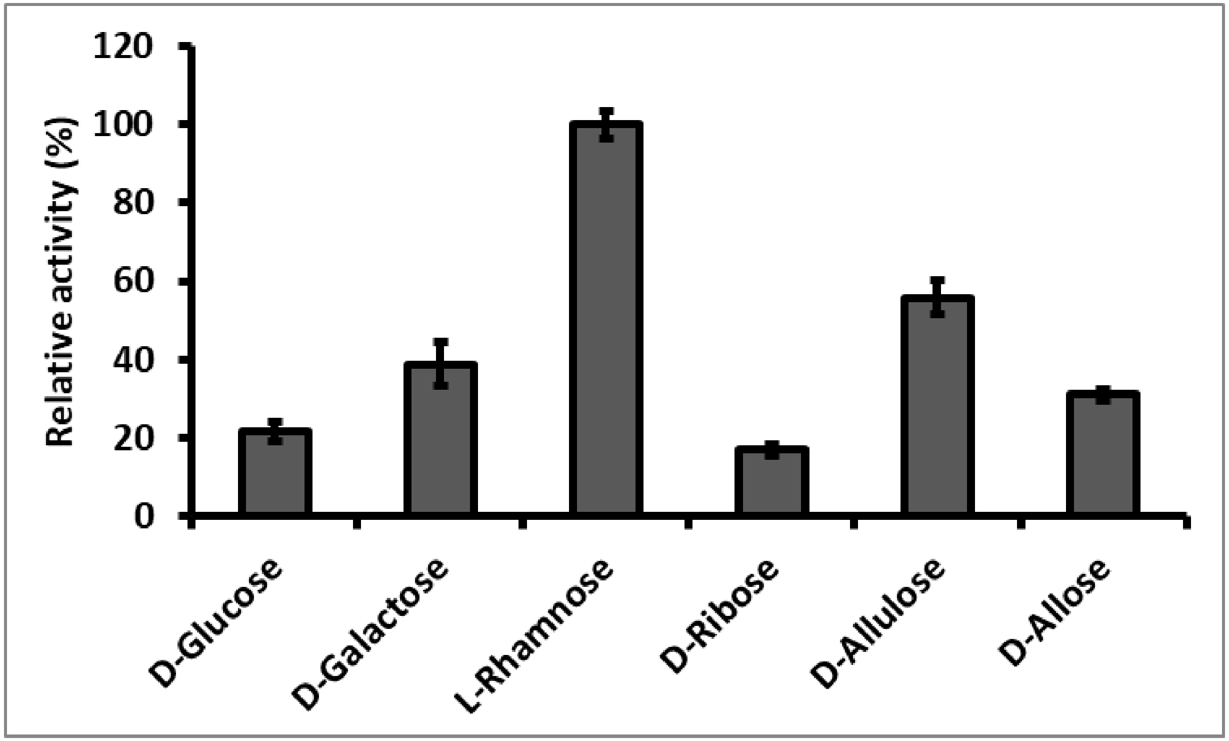

Fig. S3. Specificity of L-RI_M towards different substrates.

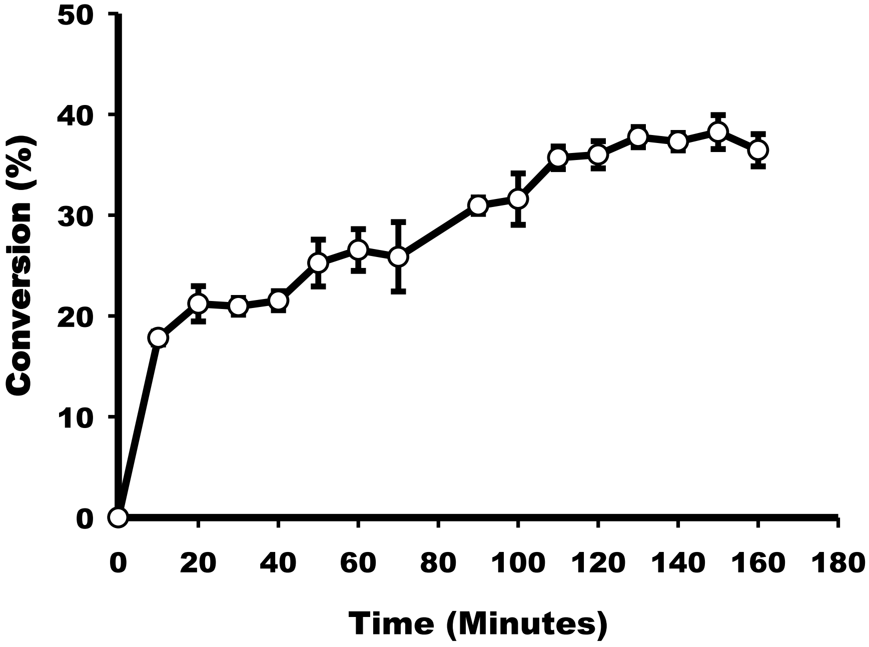

Fig. S4. Time point reaction of D-allulose (10 mM) to D-allose conversion catalyzed by L-RI_M at optimum pH and temperature conditions.

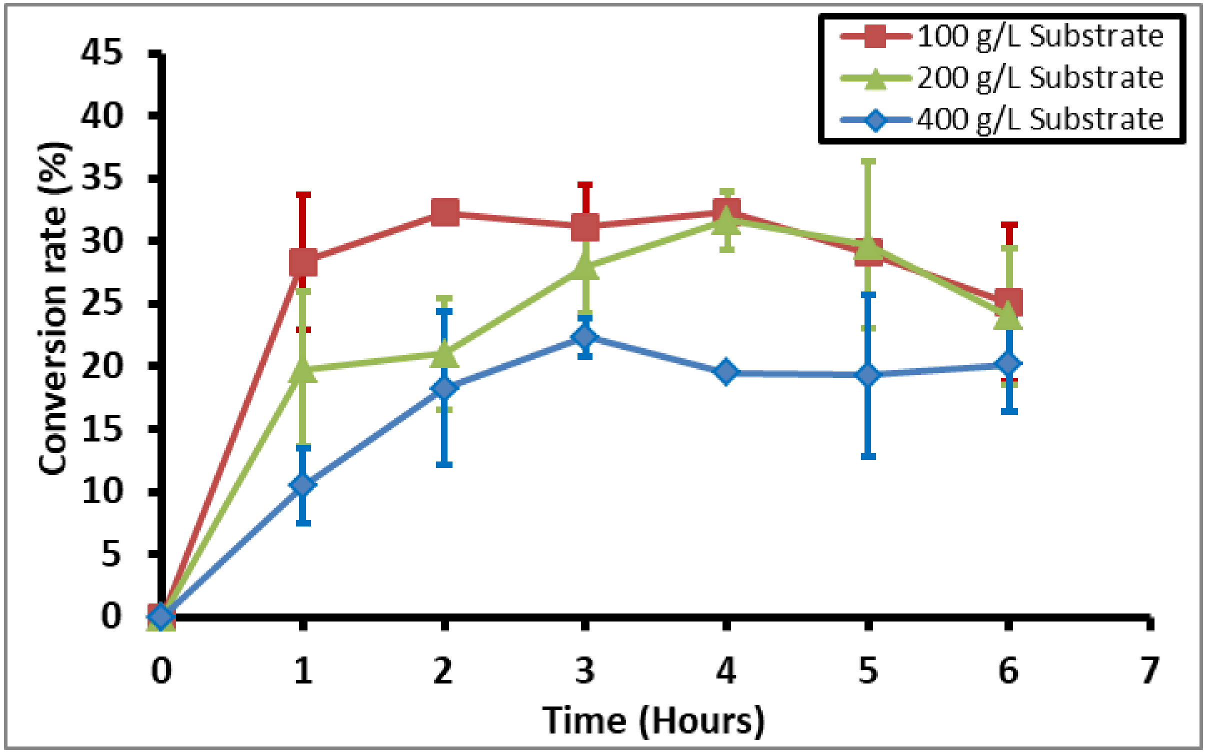

Fig. S5. Bioconversion of D-allulose into D-allose by L-RI_M with different concentrations of substrate.

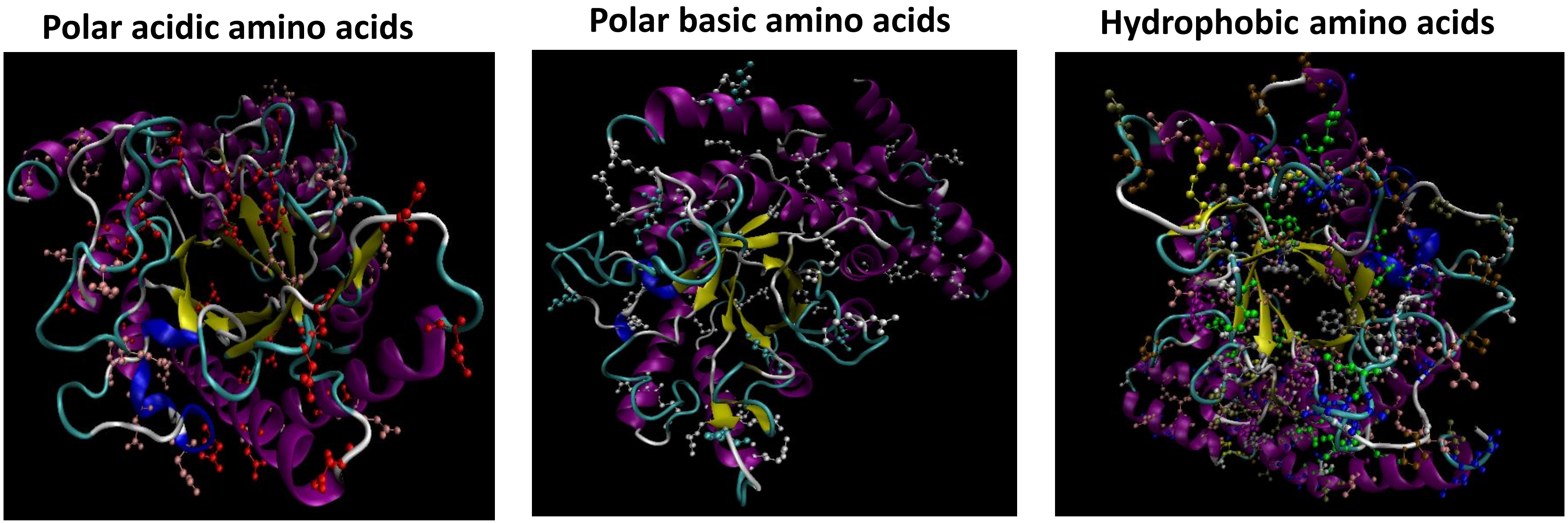

Fig. S6. Distribution of polar and non-polar amino acid residues in L-RI_M
